# Pirate Talk: Navigating Practical, Ethical, and Legal Issues Associated with Biomedical Citizen Science Interview Studies

**DOI:** 10.5334/cstp.529

**Published:** 2022-12-15

**Authors:** CHRISTI J. GUERRINI, WHITNEY BASH BROOKS, SHERYL A. MCCURDY

**Affiliations:** Baylor College of Medicine, US; Baylor College of Medicine, US; University of Texas Health Science Center, US

**Keywords:** qualitative research, DIY biology, community science, privacy, safety, terminology

## Abstract

In citizen science, in-depth interviews have advanced the understanding of project leaders’ and citizen scientists’ objectives, motivations, attitudes, and concerns. The issues encountered by researchers conducting in-depth interviews in citizen science are likely not unique to this field. However, these issues can surface and play out in distinct ways that depend on the scientific and sociopolitical circumstances of citizen science communities and projects.

Researchers’ experiences conducting in-depth interviews are the subject of a growing literature that describes considerations for conducting research with discrete populations. We aim to contribute to this literature by describing salient practical, ethical, and legal issues to consider when interviewing biomedical citizen scientists, with a focus on bottom-up biomedical citizen scientists who have loose or no affiliations with traditional scientific institutions. These issues concern how to define the interview population; earn trust and demonstrate trustworthiness given past treatment of bottom-up biomedical citizen scientists by traditional researchers and institutions; adapt research practices to the strong culture of openness that characterizes bottom-up biomedical citizen science; and manage potential safety concerns. This essay draws on our own experiences and those of other qualitative researchers and makes suggestions for addressing these issues in ways intended to protect study integrity and demonstrate respect for participants. We also identify questions that would benefit from broad input and continued study. Our objectives in sharing these lessons learned are to support future research and to improve understanding of this exciting participatory space.

## INTERVIEWING CITIZEN SCIENTISTS

In-depth interviews are a qualitative research method for collecting data about the lived experiences and perspectives of individuals and groups. The advantages of in-depth interviews include their potential for generating detailed and contextually rich information about people’s perceptions, opinions, feelings, and knowledge (Patton 2015). These data can be difficult to capture using surveys, which require the use of predetermined categories based on assumptions about participants’ answers and do not provide opportunities to follow up responses with individualized questions intended to explore or clarify (Patton 2015).

In recent years, studies that include in-depth interviews have been conducted with the goal of understanding and improving practices in citizen science. These include interviews conducted with project managers and leaders focused on study design, objectives, public engagement, and data management (Bowser et al. 2020; Kelly et al. 2019; Rambonnet et al. 2019). In-depth interviews have also been conducted with citizen scientists to understand participatory motivations, barriers, attitudes, and outcomes (Asingizwe et al. 2020; Den Broeder et al. 2017; Eveleigh et al. 2014; Everett and Geoghegan 2016; Iacovides et al. 2013; Jones et al. 2018; Merenlender et al. 2016; Raddick et al. 2010; Rotman et al. 2012).

Some interview studies have focused on citizen science projects specific to the biomedical sciences. These projects include, for example, online games designed to solve biological puzzles, digital platforms for sharing and analyzing personal health data, biological experiments conducted in community laboratories, and self-experimentation (Guerrini and Contreras 2020; Wiggins and Wilbanks 2019). Although some of these projects are led by institution-based scientists, others are developed and executed primarily or entirely by individuals working outside of traditional scientific settings who might not have relevant scientific credentials or formal training. The latter group often uses politically flavored terms to describe themselves and their activities, including do-it-yourself (DIY) biologist, community biologist, and biohacker (Trejo et al. 2021a). Consistent with a typology described by McGowan and colleagues (2017), we refer to this subgroup as bottom-up biomedical citizen scientists to emphasize their grassroots orientation. Although there are exceptions, bottom-up biomedical citizen scientists generally qualify as a hard-to-reach population because, as explained below, they can be difficult to identify and might be reluctant to engage in interview studies (Ellard-Gray et al. 2015).

Research based on in-depth interviews with bottom-up biomedical citizen scientists suggests that their goals, experiences, and concerns might differ from those of other citizen scientists. For example, in interviews with eighteen key informants from twelve organizations associated with genomic citizen science, researchers identified a shared sense of disillusionment with the goals and approaches of conventional biomedical research, as well as notable contradictions in interviewees’ enthusiasm for both communal ownership and commercialization of their research outputs (McGowan et al. 2017). In a study with managers and members of community laboratories, researchers found that a common priority of these spaces was safety, and perceived benefits of working in them included the freedom to pursue projects of personal interest, to learn new skills, and to contribute to social change (de Lange, Dunn, and Peek 2022). A third study utilizing in-depth interviews explored ethical priorities and oversight preferences of attendees at biohacking and community biology conferences and concluded that there is no one-size-fits-all solution to ethical oversight of biomedical citizen science given the diversity and independence of its communities, which one interviewee described as “a cohort of pirate ships” (Trejo et al. 2021b).

In other fields, researchers experienced in conducting in-depth interviews with hard-to-reach, hidden, or vulnerable populations have described lessons learned to support future studies (Ellard-Gray et al. 2015). As one example, a consensus group recommended that researchers conducting in-depth interviews with terminally ill patients in their homes have plans in place for, among other things, responding to requests for clinical advice and managing the presence of relatives or caregivers (Sivell et al. 2019). Focusing on a different population, researchers reflecting on an in-depth interview study conducted with African American women living with HIV emphasized ethical imperatives that included respecting participants’ choice of interview location because they are best positioned to identify spaces where they will feel safe and risks of stigma will be minimized (Fletcher et al. 2019). As a third example, researchers who had conducted in-depth interviews in conflict environments, which are plagued by unique informational, technological, and political limitations, made recommendations such as providing early and complete disclosure of researchers’ affiliations and intentions to help overcome the deep fear of exposure and distrust of outsiders that characterize these environments (Cohen and Arieli 2011).

This essay has a modest goal of contributing to this literature by describing salient practical, ethical, and legal issues that can arise when interviewing bottom-up biomedical citizen scientists. Although these issues are likely experienced in interviews with other hard-to-reach populations, they surface and play out in distinct ways that depend on the scientific and sociopolitical circumstances of biomedical citizen science communities and projects and therefore deserve attention.

Our process for selecting issues for discussion was as follows. First, we solicited insights and recommendations from six researchers (designated by I-number), including past collaborators, who have conducted in-depth interviews with bottom-up biomedical citizen scientists. They were recruited by email in November 2020; semi-structured interviews were conducted from November 2020 to January 2021 using an interview guide. Interviews were audio-recorded with permission and professionally transcribed. Transcripts were then coded and coded data were analyzed to identify categories of issues for discussion. (Additional information about interviewees can be found in Supplemental file 1: Appendix A.) Second, we reflected on the concordance of these data with our own research experiences interviewing biomedical citizen scientists and other hard-to-reach populations, including recollections of shared experiences with past interviewee-collaborators. We also reflected more generally on our research experiences for the purpose of adding nuance to or expanding on insights or suggestions.

This essay addresses select issues for consideration when planning and conducting in-depth interviews with bottom-up biomedical citizen scientists. They concern how to define the interview population; earn trust and demonstrate trustworthiness given past treatment of bottom-up biomedical citizen scientists by traditional researchers and institutions; adapt research practices to the strong culture of openness that characterizes bottom-up biomedical citizen science; and manage potential safety concerns. Drawing on other qualitative research, we make suggestions for addressing these issues in ways that are intended to protect study integrity and to demonstrate respect for participants. Along the way, we identify questions that would benefit from broad input and continued study.

## DEFINING THE INTERVIEW POPULATION

A critical step when conducting in-depth research interviews is defining the population of interest, or sample universe, consistent with the research questions (Robinson 2014). This step can be challenging given the lack of consensus on who qualifies as a biomedical citizen scientist. For example, some conceptualize biomedical citizen scientists to include life hackers experimenting with the effect of diet and other lifestyle changes on their health or well-being, users of direct-to-consumer genetic testing services and interpretation tools, and/or grinders implanting magnets and RFID chips into their bodies, while others question whether these actors fall under the citizen science umbrella.

The European Citizen Science Association identified characteristics of citizen science and published guidance that includes discussions of citizen science with health aims and citizen science conducted outside of mainstream science (ECSA 2015; ECSA 2020). Empirical studies (including our own) have attempted to translate these and other definitional supports and typologies into eligibility criteria (Borda, Gray, and Fu 2020; Follett and Strezov 2015; Guerrini et al. 2019). Still, as one researcher explained, the amount of time required to identify eligibility criteria for studies with biomedical citizen scientists should not be underestimated:

[I]t really took quite a while to get a handle on what was the population we’re actually trying to study. It wasn’t a preformed or easily identified group in either case. And so the studies ended up taking longer probably than they might otherwise if you have a known universe of participants.

(I-4)

Although not mentioned by the researcher, eligibility criteria can also impact data saturation, which is the point in data collection at which new data produce little or no additional information to address the research question (Guest, Namey, and Chen 2020). When data saturation is used to determine sample sizes in qualitative research, the heterogeneity of participant experiences and identities relevant to the research domain can increase the sample size required to reach saturation (Guest, Bunce, and Johnson 2006).

There are subgroups of biomedical citizen scientists, and some might be less difficult to define than others. For example, although there is no widely adopted model for a community laboratory, common characteristics were identified in a study to understand management of community laboratories and members’ activities and motivations (de Lange, Dunn, and Peek 2022). By contrast, it might be more challenging to identify inclusion criteria for biohackers given that biohacking encompasses a heterogenous set of identities, practices, affiliations, and values (Meyer 2021). Possible solutions to this problem include limiting participation to those who publicly self-identify as biohackers, participate in forums for biohackers, or engage in activities sometimes associated with biohacking, such as building a home laboratory.

When the interview population is defined to encompass individuals from multiple biomedical citizen science subgroups, such as community laboratories, online self-research groups, and biotechnology collaboratives, questions might arise about whether data relevant to each group should be segregated for subgroup analysis. It might be useful and even necessary to follow this analytical approach when subgroups are easily identified and subgroup differences relevant to the research questions are known, hypothesized, or indicated. However, we caution against it when subgroup boundaries are blurry or overlap or interviewees identify with multiple subgroups.

## EARNING TRUST AND DEMONSTRATING TRUSTWORTHINESS

Establishing trust is essential to all successful in-depth interviews yet can be difficult to achieve for many reasons. Distrust of academic researchers is common among bottom-up biomedical citizen scientists who view establishment science as elitist, exclusive, and self-serving, and intentionally position themselves in political or ethical opposition to traditional research institutions and practices. Distrust can also stem from a perceived tendency of academic researchers to be dismissive of the scientific contributions of bottom-up biomedical citizen scientists or unfairly critical of their activities. As one researcher explained:

I feel like gaining the trust of the community is probably the thing that differs in terms of conducting interviews with DIY versus other interviews that I’ve conducted. Because they’re more wary… [B]uilding up trust with the community, like they have to know that you’re not going to skewer them. Because there’s a lot of bioethicists, especially, who have just like written how DIY people are stupid or crazy or wrong.

(I-5)

A second researcher concurred:

[T]here’s an academic or stereotype out there … that they’re all doing these really dangerous projects. So the community as a whole, whether or not the stereotypes even exist, they’re really concerned about them and I think people don’t want to be portrayed in that way.

(I-1)

A third researcher connected wariness of outsiders to “sensationalized” media accounts of biohackers suggesting, for example, that they “are tinkering around trying to make anthrax and smallpox in their garage,” which is not the case. (I-2)

Several practices, well-known among qualitative researchers, can help researchers earn trust and demonstrate trustworthiness. First, with respect to recruitment, it is helpful to partner with respected members of the community who can inform potential interviewees about the research (Ellard-Gray et al. 2015). Second, spending unstructured time with a participant in advance of the interview can be an opportunity to develop rapport and dispel misconceptions, although some scholars warn that these interactions might frustrate the researcher’s ability to maintain objective distance during the interview (Seidman 2019). Third, emphasizing one’s institutional affiliations and professional credentials is discouraged. As one researcher explained: “[S]ort of not trying to show off your credentials or how expert you are in anything generally results in more openness and more friendly discussion.” (I-3)

Consideration should also be given to terminology and framing of issues, which qualitative researchers working with different populations have noted can be “particularly important when establishing a trusting relationship” (Plummer and Simpson 2014, p. 17). If there are questions about terms to use—for example, whether to describe the interviewee’s work as citizen science or biohacking—a good approach is simply to ask the interviewee their preference. Additionally, one researcher recommended giving special attention to balance of questions. For example, questions about potential activity risks should be accompanied by questions about potential activity benefits. The researcher observed that some traditional scholars “are maybe guilty of this—of assuming that there are some concerns, or something negative, without having the equivalent question to gauge what the positive is. … And so that really strikes [citizen scientists] who feel that there is something very positive about what they’re doing.” (I-5)

Finally, researchers should give careful consideration to whether and how they will offer compensation to interviewees, which can have implications for trust. In any research endeavor, and assuming sufficient funding, it can be difficult to identify what kind and level of compensation, if any, is appropriate and not coercive or unduly influential (Gelinas et al. 2018). In interview studies with biomedical citizen scientists, some of us elected to compensate interviewees for their time at the same rate that we compensate domain experts and key stakeholders as a demonstration of respect (Guerrini et al. 2022; Trejo et al. 2021b). However, others might disagree with this approach, and we encourage continued examination of the relational impacts and ethical implications of reimbursement for interviews with biomedical citizen scientists to help guide future research.

## NAVIGATING A CULTURE OF OPENNESS

A defining feature of biomedical citizen science is a commitment to openness of processes, data, and other outputs—and, in some cases, what has been described as “radical openness” that encompasses shared ownership and deinstitutionalization (McGowan et al. 2017, p. 505)—and this commitment manifests in almost every aspect of in-depth interviews with bottom-up biomedical citizen scientists. First, although promises of confidential participation have been described as “an essential qualitative research ethics assurance” (Tolich and Tumilty 2020, p. 20) and is the norm for many interview studies approved by institutional review boards (IRBs) (Seidman 2019), our experience has been that many bottom-up biomedical citizen scientists are agnostic about being associated by name with their interview data. Similar to some narrators in oral history projects (Yow 2015), some biomedical citizen scientist interviewees might even prefer to go on the record.

Other researchers described similar experiences with biomedical citizen scientists who “openly said that they wanted [to use] their names.” (I-6) One explained how their team asked interviewees “‘Do you want to be identified or not?’ And every single one said yes.” (I-4) Reflecting on all of the IRB-approved interview studies in which they had been involved, the researcher emphasized the rarity of this response: “I don’t think I’ve experienced that with other studies. With other studies, it’s sort of just taken for granted that you’ll be de-identified, and no one will know that you participated, and accepted that that will be the case.” (I-4)

Second, biomedical citizen scientists’ commitment to openness manifests in a general willingness to speak freely about most topics—few appear to be sensitive or out of bounds—and about each other. There is a strong culture in some DIY biology and biohacker communities in particular of publicly calling out those perceived to be engaged in unsafe or scientifically unsound practices or otherwise acting inconsistent with community norms (including norms of openness), and that culture can be salient in interviews. Such remarks are useful in contextualizing other information shared by the interviewee, and when the study design includes recruitment by snowball sampling, in identifying interviewees with potentially very different perspectives on the research questions.

Third, openness can manifest during interviews as vulgar language. Explained one researcher:

[A]nyone who’s coming into this community to ask questions should not have sensitive ears. … There’s a lot of profanity and generally there tends to be language that you don’t hear in a boardroom and just more like what you would hear on a pirate ship.

(I-3)

This issue is not unique to bottom-up biomedical citizen science, and as reported elsewhere, problems can arise when quotations in manuscripts include, among other things, swear words that offend publishers, reviewers, or readers (Corden and Sainsbury 2006a,b). In a recent manuscript, some of us quoted interview data that included what might be described as vulgar language because it supported key findings (Trejo et al. 2021b). Although the profanities were not gratuitous but in service to the points being made, there was concern that their inclusion might be distracting or offensive. However, we elected not to censor the data and explained our reasoning in a note to editors, who did not object to our decision.

Fourth, a culture of openness, as well as commitments to democratic approaches to scientific knowledge production and community-focused forms of research, can manifest via expectations of and explicit requests for access to study materials, data, and findings. There is growing recognition that researchers have ethical obligations to return research results or other benefits to participants (Botkin et al. 2018), but this current appears to run especially strong in biomedical citizen science communities that emphasize reciprocity. Indeed, participants expressed concern to one researcher “with doing all of these time-consuming interviews and then not seeing any benefit for the community.” (I-1) At a minimum, qualitative researchers should address these concerns by aiming to publish their findings in open-access formats. Given that open-access publication fees can run thousands of dollars per manuscript, budget plans will need to account for those fees. To further broaden reach and accessibility, researchers might also consider translating findings for non-academic audiences into infographics and brief summaries for broad dissemination (Ross-Hellauer et al. 2020).

Further, researchers should expect some participants to request study materials, such as interview guides and coding schemes. In general, researchers have discretion to share requested materials with participants so long as doing so does not violate their IRB-approved protocol. When interviewees are bottom-up biomedical citizen scientists, however, decisions to share requested materials should account for the possibility that requestors will disclose them to others, including by publishing them on the internet before a planned release date or in a manner that could disrupt future research activities. This was, in fact, the distressing experience of one researcher: “[I]t was really disappointing because I felt like it broke the trust that we had developed with this participant, that he would share our research materials in that way.” (I-4) Nevertheless, the researcher had come to understand the requestor’s action as faithful to the objectives of citizen science and learned from the experience that, when interviewing bottom-up biomedical citizen scientists in particular, challenges to research questions and what might be considered standard and uncontroversial methods should be expected:

But it also helped me to realize that the social contract between researchers and participants is necessarily going to be disrupted when you’re working with people who aren’t in the traditional research spaces. And so it was a really good lesson. … [T]hat perhaps these people whose perspectives we think are really valuable are actively trying to disrupt the way that science happens. And … that we might get pushback for the way that we do our studies or how we’re framing the questions or the problems. And that we should be open to that. That there are going to be people who, from a scientific perspective, might gum up the works for us. But in fact they’re actually just pushing the envelope.

(I-4)

## MANAGING CONCERNS ABOUT SAFETY

The last consideration is safety. As described above, biomedical citizen science is diverse, and many activities, such as aggregating health data for secondary study or practicing gene editing of yeast genomes in community laboratories, do not present safety issues for participants, bystanders, or the environment. However, some biomedical citizen scientists are engaged in self-experimentation that poses some risk of self-harm, such as infection at injection sites, or laboratory research involving equipment, materials, or byproducts that, if mishandled, might harm not only the participants but also their neighbors or have adverse impacts on ecosystems.

Projects involving institution-based scientists are often required to undergo IRB review to ensure that risks to human research subjects are minimized and reasonable in relation to benefits. Otherwise, biomedical citizen scientists have demonstrated their commitment to safety in a number of ways (Wexler et al. 2022) that include co-development of a 250-page Community Biology Biosafety Handbook (Armendariz et al. 2020). Many community laboratories have procedures in place to ensure that research conducted on their premises is low risk and complies with established biosafety standards (Guerrini, Spencer, and Zettler 2019); in 2019, members of some community laboratories participated in a three-day biosafety training program (Baltimore Under Ground Science Space n.d.). Further, self-experimenters and individuals working in home laboratories have explained that they conduct research, consult with peers, and engage in ethical self-reflection before proceeding with projects (Trejo et al. 2021b).

Yet, even the most conscientious citizen scientist might not be aware of all of the risks associated with their activities or the regulations and practice standards intended to minimize those risks. Ethical concerns and liability questions arise when researchers become aware of activities, possibly prohibited, that risk harm to interviewees or the environment. One researcher, for example, learned that a citizen science group had future plans that “created a sort of ethical conundrum for us about what are our obligations as researchers to try to educate our participants if we think they might be getting themselves into some big messes down the road.” (I-4) In rare circumstances, researchers might be legally obligated to report interviewees’ activities (Seidman 2019), or as an ethical matter, they might have a duty of responsible citizenship to do so (Yow 2015). If legally compelled to produce records that include evidence of wrongdoing, researchers will find themselves in ethical conflict with their obligation to the welfare of the participant as well as in breach of any promises to protect the participant’s identity (Seidman 2019).

Such dilemmas are not unique to bottom-up biomedical citizen science (McCurdy and Ross 2018) and ultimately “must be solved in their context” (Yow 2015, p. 151). Guillemin and Gillam (2004, p. 276) describe the practice of reflexivity to sensitize researchers to these and other “ethically important moments” that manifest during the “ordinary, everyday” conduct of research. The practice of reflexivity also promotes anticipation of, and the development of skills and tools to manage and perhaps even preempt, problematic situations. We encourage further conversation around what preemptive and responsive plans related to safety issues raised during interviews with bottom-up biomedical citizen scientists might look like and how such plans might be disclosed to participants during the informed consent process. For example, researchers might explain in consent documents how they will manage interview data describing risks of serious harm. Guidance on these issues, with input from IRBs and citizen scientists, will not only provide needed clarity for researchers, but also help ensure that interviewees’ consent to participate is fully informed.

## CLOSING REMARKS

In-depth interviews with biomedical citizen scientists can yield important information about the past, present, and future of participant-driven biomedical discovery and innovation. They can elucidate the conditions under which these activities will emerge and flourish and provide insight on the technical, practical, ethical, and regulatory supports and issues that are encountered along the way. More generally, in-depth interview studies with biomedical citizen scientists can help test and refine conceptual frameworks relevant to the motivations for, facilitators of, and barriers to public participation in science.

Our intention in sharing experiences with using in-depth interview methods in this exciting participatory space is to support research on these and other questions. Critically, this research should be sensitive to the priorities and concerns that interview participants associate with their biomedical citizen science work and communities. To help achieve this objective, we recommend future engagement with biomedical citizen scientists on the issues addressed in this essay as well as attention to the innovative work that some are doing on matters of governance, ethics, and safety (Kuiken 2020; Pearlman and Kong 2021; Wexler et al. 2022), which might provide additional insight into stakeholder perspectives.

## Supplementary Material

Supplemental File
