# Supplementary material for: Pirate Talk: Navigating Practical, Ethical, and Legal Issues Associated with Biomedical Citizen Science Interview Studies
Source: Citiz Sci. Author manuscript; Available in PMC 2023 Jun 2. (PMC10238071; doi:10.5334/cstp.529)
Supplement: Supplemental File [file NIHMS1899288-supplement-Supplemental_File.pdf]

Appendix A. Interviewee-researcher characteristics

Non-identifying characteristics of interviewee-researchers are as follows. Five resided in the United States at the time of their interview, and all were affiliated with academic institutions as trainees, consultants, or employees at the time of their participation in biomedical citizen scientist interview studies. The qualitative training of four interviewee-researchers included at least graduate school coursework or other academic training programs; three interviewee-researchers also or alternatively were trained in connection with professional employment. All but one interviewee-researcher had participated in interviews involving not only biomedical citizen scientists, but also other populations, and so were able to compare experiences. The total number of studies involving in-depth interviews that interviewee-researchers had participated in ranged from one to fifteen. Most had participated in four to six.
